# The cancer incidence and mortality among children and adolescents during the period of 2010‐2014 in Henan Province, China

**DOI:** 10.1002/cam4.1952

**Published:** 2019-01-18

**Authors:** Qiong Chen, Zhen Guo, Shuzheng Liu, Peiliang Quan, Xiaoqin Cao, Lanwei Guo, Shaokai Zhang, Xibin Sun

**Affiliations:** ^1^ Department of Epidemiology Affiliated Cancer Hospital of Zhengzhou University/Henan Provincial Cancer Hospital Zhengzhou China; ^2^ Department of Laboratory Medicine Henan Provincial Chest Hospital Zhengzhou China

**Keywords:** childhood cancer, Henan, incidence and mortality, leukemia, population‐based cancer registry

## Abstract

**Objective:**

The cancer etiology in children and adolescents is largely different with that in adults, and the description in epidemiology still remains deficiency. Therefore, we described the cancer incidence and related epidemiological features in children and adolescents to provide clues for etiological studies.

**Methods:**

Cancer incidence stratified by age, gender, and areas was calculated using data extracted from population‐based cancer registries in Henan Province, China. All cancer among children aged 0‐19 years were reclassified according to category criteria of the International Classification of Childhood Cancer, 3rd Edition (ICCC‐3). Age‐standardized rate (WSR) was calculated using Segi's world standardized population by the direct method, and it was expressed per million person‐years.

**Results:**

The crude cancer incidence and mortality were 87.56 and 36.32 per million person‐years among children aged 0‐19 years, and the WSRs slightly changed compared with crude incidence and mortality, and they were 87.36 and 35.46 per million person‐years. Leukemia and central nervous system neoplasms (CNS) were the most common cancer categories both in children aged 0‐14 years and in adolescents aged 15‐19 years in regardless of gender and areas. Tiny difference of incidence and mortality existed in different age groups across 0‐14 years; however, they were higher in adolescents aged 15‐19 years than that in children aged 0‐14 years. Among children aged 0‐19 years, the cancer incidence and mortality were predominant in boys, and the sex ratio was 1.19; however, it was varied by diagnostic categories.

**Conclusion:**

This is the first study that described the cancer incidence and mortality among children aged 0‐19 in Henan Province, and it would help researchers to understand the burden and epidemiological characteristics of childhood cancer, and hence suggested clues for the etiological studies.

## 1 INTRODUCTION

Childhood cancer refers to cancer that occurs in children aged 0‐19 years old.^1^, ^2^ According to GLOBOCAN 2018, the childhood cancer usually accounted for 1% of the total cancer,^3^ and the incidence ranging from 50 to 180 per million person‐years with the highest value in Southern Europe, and the lowest value in sub‐Saharan Africa.^1^, ^3^ Although the cancer incidence in children and adolescents was low, however, the diagnosis of cancer is a life‐altering event for them as well as their families. Furthermore, the childhood cancer incidence was increasing in all regions of the world except sub‐Saharan Africa.^1^, ^2^ Although advances in treatment have increased the five‐year survival rate, however, it still be the second leading cause of death in developed countries.^4^, ^5^ In developing countries, the emergence of cancer in children was thought as greater public health problem than in the past along with the control of communicable diseases.^6^, ^7^

The etiology of childhood cancer largely remains unclear until now, and the description and inspection of the epidemiological features of particular neoplasms may provide insights into their etiology, and clues to the methods for prevention. However, the comparisons between geographical areas, subgroups population, and over time have proved to be more difficult to undertake for children than for adults due to the low incidence and classification by morphology and behavior.^1^, ^8^ Nationwide in China, only one study estimated the incidence, mortality, and survival among children aged 0‐14 years, and it found that the overall WSR incidence and mortality were 87.1 and 36.3 per million person‐years; overall 5‐year relative survival reached 71.9%.^9^ Data in some cities including Beijing, Guangzhou, Dalian, Zhongshan, and Hong Kong were also presented in IARC publications.^10^ However, the descriptive data in cancer among children, especially at the provincial level, are still lacking. Therefore, we present statistical result using the population‐based cancer registration data of Henan Province which have the biggest population in China.

## 2 MATERIALS AND METHODS

### 2.1 Data source and quality control

Henan Provincial Cancer Registry is responsible for the collection, evaluation, and management of cancer data in Henan, China, and Cancer Registration Database was also established. The data were collected by the county level cancer registries, which reported data annually to the Provincial Cancer Registry. The sources of cancer diagnoses that reported to local registries including hospitals, health insurance system, new rural cooperative medical system, and vital statistical system. Cancer diagnoses were coded according to topography, morphology, and behavior using the International Classification of Diseases for Oncology, 3rd Edition (ICD‐O‐3) and the International Statistical Classification of Diseases and Related Health Problems 10th Revision (ICD‐10). The validity of cancer cases was checked using the data criteria of Cancer Incidence in Five Continents to detect unlikely or impossible codes, or combinations of codes. These cases were then sent back to data source unit by local registries to verify the reality of data, and the registrars updated the results in the cancer registration database. The database included data from 17 registries in 2010 and 2011, 20 registries in 2012 and 2013, and 27 registries in 2014, which were evaluated based on the criteriaof “Guideline for Chinese Cancer Registration”^11^ and International Agency for Research on Cancer/International Association of Cancer Registries (IARC/IACR),^12^ and it was accepted by the National Cancer Registry Annual Report in China. The validity, reliability, completeness, and comparability of cancer registry data were evaluated based on a comprehensive consideration of a series of indexes including the mortality to incidence (M/I) ratio, the percentage of cases morphologically verified (MV, %), the percentage of death certificate‐only cases (DCO, %), the percentage of the diagnosis of unknown basis (UB, %), and the stability of cancer trends over years. The data quality indices including M/I, MV%, DCO%, and UB% in Henan Cancer Registries during 2010‐2014 were 0.64, 67.94%, 2.35%, and 0.28%, respectively. The total cancer incidence and mortality were stable during the calendar years 2010‐2014.

Cancer mortality data were derived from the vital statistics in local registries. The population data in each cancer registry were provided by the local Bureau of Statistics in area where each cancer registry covered, and person‐years were defined as the sum of the population counts for a registry in each year from 2010 to 2014, categorized by sex, age, and areas.

The geographical distribution of the 27 cancer registries was evenly, and they equally located in the East, South, West, and North of Henan Province, China. The population covered by the 27 cancer registries in 2014 was 21 044 835 including 10 851 503 males and 10 193 332 females, and it accounted for 19.73% of the total population in Henan Province, China.^13^

In our study, Cancer incidence data among children aged 0‐19 years during the calendar years between 2010 and 2014 were extracted from the cancer incidence and mortality database managed by Henan Provincial Cancer Registry. Our study was approved by the Ethics Committee of Henan Cancer Hospital, and it conforms to the provisions of the Declaration of Helsinki.

### 2.2 Statistical analysis

All statistical analyses were conducted using SAS 9.4 version (SAS Institute, Cary, NC). All childhood cancer was reclassified according to category criteria of the International Classification of Childhood Cancer, 3rd Edition (ICCC‐3)^14^ which was based on the morphology and topography codes used in ICD‐O‐3, and the ICCC‐3 names were used as cancer categories. All areas covered by cancer registries were classified into urban or rural areas according to the National Bureau of Statistics of the People's Republic of China. Age‐specific rates (ASR) for four 5‐year age groups were calculated, and crude cancer rates among children (aged 0‐14 years), adolescents (aged 15‐19 years), and in total cases aged 0‐19 years stratified by gender and area (urban/rural) were also calculated. For comparison with data worldwide, age‐standardized rates (WSRs) for the 0‐14 years and 0‐19 years age groups were calculated via direct method using the Segi's standardized population with the weights 12, 10, 9, and 9 for the four age groups 0‐4 years, 5‐9 years, 10‐14 years, and 15‐19 years, respectively.^15^ The incidence sex ratios were calculated by dividing the incidence in male individuals with that in female individuals.

## 3 RESULTS

### 3.1 Person‐years

The person‐years covered by cancer registries in each age group stratified by areas and gender were shown in Table 1, in total, there were 22 248 098 person‐years in all cases aged 0‐19 years, and the figure in children aged 0‐14 and adolescents aged 15‐19 years old were 16 412 890 and 5 835 208, respectively. Among children, there were 8 914 374 and 7 498 516 person‐years in boys and girls, respectively, and the numbers in city and rural areas were 2 143 940 and 14 268 950, respectively. Among adolescents, there were 3 068 021 and 2 767 187 person‐years in boys and girls, and the numbers in city and rural areas were 818 073 and 5 017 135, respectively.

**Table 1:** Person‐years covered by cancer registries in each age group stratified by gender and areas during 2010‐2014 in Henan, China

Age group	Gender		Areas		Total
	Male	Female	City area	Rural area	
0	529 129	454 815	119 184	864 760	983 944
1‐4	2 450 459	2 021 368	604 004	3 867 823	4 471 827
5‐9	2 942 630	2 484 896	728 403	4 699 123	5 427 526
10‐15	2 992 156	2 537 437	692 349	4 837 244	5 529 593
15‐19	3 068 021	2 767 187	818 073	5 017 135	5 835 208
0‐14	8 914 374	7 498 516	2 143 940	14 268 950	16 412 890

### 3.2 Cancer incidence

As shown in Table 2, during 2010‐2014, 1948 cases aged 0‐19 years were diagnosed with cancer among which there were 1396 children and 552 adolescents, and they accounted for 0.97% of cancer in all age groups. The crude incidence in children was 85.06 per million person‐years, it was 94.60 in adolescents, and in total, it was 87.56 per million person‐years. The WSR incidence in cases aged 0‐19 years and children aged 0‐14 years were slightly changed after standardized by Segi's standardized population. The most common cancer categories in children were in line with that in adolescents, which were leukemia, central nervous system (CNS) neoplasms, carcinoma and melanoma, and they approximately accounted for 45%, 15%, and 5%, respectively.

**Table 2:** The incident cases number and incidence rate of childhood cancer during 2010‐2014 in Henan Province, China

ICCC3 category^a^		Incident cases number				Proportion (%)		Age‐specific incidence^b^				Crude rate		WSR^c^	
		0‐4	5‐9	10‐14	15‐19	0‐14	15‐19	0‐4	5‐9	10‐14	15‐19	0‐14	0‐19	0‐14	0‐19
I	Leukemia	248	233	210	200	49.50	36.23	45.46	42.93	37.98	34.27	42.10	40.05	42.47	40.63
a.	Lymphoid	52	40	48	49	10.03	8.88	9.53	7.37	8.68	8.40	8.53	8.50	8.59	8.54
b.	Acute myeloid	19	30	8	18	4.08	3.26	3.48	5.53	1.45	3.08	3.47	3.37	3.55	3.45
c.	CMD	2	2	1	3	0.36	0.54	0.37	0.37	0.18	0.51	0.30	0.36	0.31	0.36
d.	MDS & other	7	15	8	13	2.15	2.36	1.28	2.76	1.45	2.23	1.83	1.93	1.81	1.90
e.	Unspecified	168	146	145	117	32.88	21.20	30.79	26.90	26.22	20.05	27.97	25.89	28.21	26.37
II	Lymphoma & related	15	22	23	26	4.30	4.71	2.75	4.05	4.16	4.46	3.66	3.87	3.58	3.78
a.	Hodgkin	1	6	6	8	0.93	1.45	0.18	1.11	1.09	1.37	0.79	0.94	0.74	0.88
b.	Non‐Hodgkin except BL	5	7	9	7	1.50	1.27	0.92	1.29	1.63	1.20	1.28	1.26	1.24	1.23
c.	Burkitt (BL)	0	0	0	0	0.00	0.00	0.00	0.00	0.00	0.00	0.00	0.00	0.00	0.00
d.	Lymphoreticular	0	1	0	0	0.07	0.00	0.00	0.18	0.00	0.00	0.06	0.04	0.06	0.05
e.	Unspecified	9	8	8	0	1.79	0.00	1.65	1.47	1.45	1.89	1.52	1.62	1.53	1.61
III	CNS neoplasms	69	89	89	75	17.69	13.59	12.65	16.40	16.10	12.85	15.05	14.47	14.86	14.41
a.	Ependymoma	0	0	0	1	0.00	0.18	0.00	0.00	0.00	0.17	0.00	0.04	0.00	0.04
b.	Astrocytoma	5	13	7	7	1.79	1.27	0.92	2.40	1.27	1.20	1.52	1.44	1.49	1.43
c.	CNS embryonal	0	4	3	0	0.50	0.00	0.00	0.74	0.54	0.00	0.43	0.31	0.40	0.31
d.	Other gliomas	13	15	15	10	3.08	1.81	2.38	2.76	2.71	1.71	2.62	2.38	2.60	2.40
e.	Other specified	4	6	8	6	1.29	1.09	0.73	1.11	1.45	1.03	1.10	1.08	1.06	1.05
f.	Unspecified CNS	47	51	56	51	11.03	9.24	8.61	9.40	10.13	8.74	9.38	9.21	9.31	9.18
IV	Neuroblastoma	3	0	2	0	0.36	0.00	0.55	0.00	0.36	0.00	0.30	0.22	0.32	0.25
a.	(Ganglio)neuroblastoma	3	0	0	0	0.21	0.00	0.55	0.00	0.00	0.00	0.18	0.13	0.21	0.16
b.	Peripheral nervous	0	0	2	0	0.14	0.00	0.00	0.18	0.00	0.00	0.79	0.09	0.11	0.08
V	Retinoblastoma	12	1	0	0	0.93	0.00	2.20	0.18	0.00	0.00	0.79	0.58	0.91	0.71
VI	Renal tumors	24	7	6	2	2.65	0.36	4.40	1.29	1.09	0.34	2.25	1.75	2.43	1.96
a.	Nephroblastoma	11	5	0	0	1.15	0.00	2.02	0.92	0.00	0.00	0.97	0.72	1.08	0.84
b.	Renal carcinoma	2	1	3	1	0.43	0.18	0.37	0.18	0.54	0.17	0.37	0.31	0.36	0.32
c.	Unspecified	11	1	3	1	1.07	0.18	2.02	0.18	0.54	0.17	0.91	0.72	1.00	0.81
VII	Hepatic tumors	6	1	5	16	0.86	2.90	1.10	0.18	0.90	2.74	0.73	1.26	0.75	1.20
a.	Hepatoblastoma	5	0	0	0	0.36	0.00	0.92	0.00	0.00	0.00	0.30	0.22	0.35	0.27
b.	Hepatic carcinoma	0	0	3	8	0.21	1.45	0.00	0.00	0.54	1.37	0.18	0.49	0.16	0.43
c.	Unspecified	1	1	2	8	0.29	1.45	0.18	0.18	0.36	1.37	0.24	0.54	0.24	0.49
VIII	Bone tumors	7	19	43	54	4.94	9.78	1.28	3.50	7.78	9.25	4.20	5.53	3.88	5.09
a.	Osteosarcoma	0	5	20	19	1.79	3.44	0.00	0.92	3.62	3.26	1.52	1.98	1.35	1.78
b.	Chondrosarcoma	0	0	5	9	0.36	1.63	0.00	0.00	0.90	1.54	0.30	0.63	0.26	0.55
c.	Ewing & related	0	0	0	0	0.00	0.00	0.00	0.00	0.00	0.00	0.00	0.00	0.00	0.00
d.	Other specified	0	1	2	2	0.21	0.36	0.00	0.18	0.36	0.34	0.18	0.22	0.16	0.20
e.	Unspecified	7	13	16	24	2.58	4.35	1.28	2.40	2.89	4.11	2.19	2.70	2.11	2.56
IX	Soft tissue sarcoma	9	16	8	12	2.36	2.17	1.65	2.95	1.45	2.06	2.01	2.02	2.01	2.02
a.	Rhabdomyosarcoma	1	2	1	0	0.29	0.00	0.18	0.37	0.18	0.00	0.24	0.18	0.24	0.19
b.	Fibrosarcoma	0	1	0	0	0.07	0.00	0.00	0.18	0.00	0.00	0.06	0.04	0.06	0.05
c.	Kaposi sarcoma	0	0	0	0	0.00	0.00	0.00	0.00	0.00	0.00	0.00	0.00	0.00	0.00
d.	Other specified	7	10	7	12	1.72	2.17	1.28	1.84	1.27	2.06	1.46	1.62	1.46	1.59
e.	Unspecified	1	3	0	0	0.29	0.00	0.18	0.55	0.00	0.00	0.24	0.18	0.25	0.19
X	Germ cell tumors	10	6	9	29	1.79	5.25	1.83	1.11	1.63	4.97	1.52	2.43	1.54	2.31
a.	CNS germ cell	0	1	0	1	0.07	0.18	0.00	0.18	0.00	0.17	0.06	0.09	0.06	0.08
b.	Other extragonadal	4	0	0	1	0.29	0.18	0.73	0.00	0.00	0.17	0.24	0.22	0.28	0.26
c.	Gonadal germ cell	4	0	0	13	0.29	2.36	0.73	0.00	0.00	2.23	0.24	0.76	0.28	0.72
d.	Gonadal carcinoma	1	3	7	8	0.79	1.45	0.18	0.55	1.27	1.37	0.67	0.85	0.62	0.79
e.	Unspecified gonadal	1	2	2	6	0.36	1.09	0.18	0.37	0.36	1.03	0.30	0.49	0.29	0.46
XI	Carcinoma & melanoma	18	26	30	72	5.30	13.04	3.30	4.79	5.43	12.34	4.51	6.56	4.40	6.18
a.	Adrenocortical	2	1	0	0	0.21	0.00	0.37	0.18	0.00	0.00	0.18	0.13	0.20	0.16
b.	Thyroid	0	4	4	12	0.57	2.17	0.00	0.74	0.72	2.06	0.49	0.90	0.45	0.81
c.	Nasopharyngeal	0	0	1	3	0.07	0.54	0.00	0.00	0.18	0.51	0.06	0.18	0.05	0.16
d.	Melanoma	14	5	2	5	1.50	0.91	2.57	0.92	0.36	0.86	1.28	1.17	1.40	1.27
e.	Skin carcinoma	0	2	2	8	0.29	1.45	0.00	0.37	0.36	1.37	0.24	0.54	0.22	0.48
f.	Other & unspecified	2	14	21	44	2.65	7.97	0.37	2.58	3.80	7.54	2.25	3.64	2.08	3.31
XII	Other & unspecified	54	41	35	66	9.31	11.96	9.90	7.55	6.33	11.31	7.92	8.81	8.11	8.83
a.	Other specified	0	0	0	0	0.00	0.00	0.00	0.00	0.00	0.00	0.00	0.00	0.00	0.00
b.	Other unspecified	54	41	35	66	9.31	11.96	9.90	7.55	6.33	11.31	7.92	8.81	8.11	8.83
	Total	475	461	460	552	100.00	100.00	87.06	84.94	83.19	94.60	85.06	87.56	85.25	87.36

a Childhood Cancer Categorized by International Classification of Childhood Cancer, Third Edition, ICCC3.

b The age‐specific incidence (ASR) and crude rate were calculated by per million person‐years.

c The age‐standardized rate was adjusted by World Segi's standardized population (WSR).

### 3.3 Age‐specific cancer incidence in children and adolescents

As shown in Table 2, among children aged 0‐14 years, slightly difference of cancer incidence existed in different age groups, and it was peaked at 0‐4 years age group, followed by 5‐9 years, and 10‐14 years groups with the incidence of 87.06, 84.94, and 83.19 per million person‐years, respectively. Cancer incidence in adolescents aged 15‐19 years was 94.60 per million person‐years which was higher than that in children aged 0‐14 years. Among the different cancer diagnostic categories, leukemia and CNS neoplasms were the most common across all age groups in children and adolescents. The third common cancer was different in different age groups with renal tumor in 0‐4 years age groups, bone tumors in 10‐14 years age group, and carcinoma and melanoma in 5‐9 years age groups and in adolescents.

### 3.4 Sex difference of cancer incidence in children and adolescents

As shown in Tables 3 and 4, in total, the cancer incidence in cases aged 0‐19 years was higher in boys than that in girls, it was 94.39 and 79.59 per million person‐years in boys and girls, and the sex ratio was 1.19. The sex ratio was higher in children aged 0‐14 years than that in adolescents aged 15‐19 years with the value 1.24 and 1.07, respectively. It was also varied by diagnostic categories. The bone tumors had the highest sex ratio which was 1.59, followed by CNS neoplasms and lymphoma and related which were 1.58 and 1.52, respectively. In some cancer categories such as germ cell tumors and retinoblastoma, it showed a characteristic that predominant in girls and the sex ratio were 0.19 and 0.73, respectively.

**Table 3:** The incident cases number and incidence rate of childhood cancer during 2010‐2014 in Henan Province, China, stratified by gender

Gender	ICCC3 category^a^		Incident cases number				Proportion (%)		Age‐specific incidence^b^				Crude rate		WSR^c^	
			0‐4	5‐9	10‐14	15‐19	0‐14	15‐19	0‐4	5‐9	10‐14	15‐19	0‐14	0‐19	0‐14	0‐19
Boys	I	Leukemia	136	140	117	121	47.29	40.33	45.64	47.58	39.10	39.44	44.09	42.90	44.37	43.26
	II	Lymphoma & related	10	14	14	17	4.57	5.67	3.36	4.76	4.68	5.54	4.26	4.59	4.19	4.50
	III	CNS neoplasms	43	58	64	44	19.86	14.67	14.43	19.71	21.39	14.34	18.51	17.44	18.15	17.30
	IV	Neuroblastoma	1	0	2	0	0.36	0.00	0.34	0.00	0.67	0.00	0.34	0.25	0.32	0.25
	V	Retino blastoma	6	0	0	0	0.72	0.00	2.01	0.00	0.00	0.00	0.67	0.50	0.78	0.60
	VI	Renal tumors	15	3	6	0	2.89	0.00	5.03	1.02	2.01	0.00	2.69	2.00	2.86	2.22
	VII	Hepatic tumors	3	1	3	9	0.84	3.00	1.01	0.34	1.00	2.93	0.79	1.34	0.79	1.27
	VIII	Bone tumors	2	12	31	35	5.42	11.67	0.67	4.08	10.36	11.41	5.05	6.68	4.58	6.12
	IX	Soft tissue sarcoma	6	7	6	8	2.29	2.67	2.01	2.38	2.01	2.61	2.13	2.25	2.13	2.24
	X	Germ cell tumors	4	2	1	3	0.84	1.00	1.34	0.68	0.33	0.98	0.79	0.83	0.84	0.87
	XI	Carcinoma & melanoma	10	20	19	31	5.90	10.33	3.36	6.80	6.35	10.10	5.50	6.68	5.34	6.41
	XII	Other & unspecified	32	20	23	32	9.03	10.67	10.74	6.80	7.69	10.43	8.41	8.93	8.58	9.00
		Boys total	268	277	286	300	100.00	100.00	89.95	94.13	95.58	97.78	93.22	94.39	92.93	94.02
Girls	I	Leukemia	112	93	93	79	52.74	31.35	45.23	37.43	36.65	28.55	39.74	36.72	40.22	37.60
	II	Lymphoma & related	5	8	9	9	3.89	3.57	2.02	3.22	3.55	3.25	2.93	3.02	2.85	2.94
	III	CNS neoplasms	26	31	25	31	14.51	12.30	10.50	12.48	9.85	11.20	10.94	11.01	10.95	11.01
	IV	Neuroblastoma	2	0	0	0	0.35	0.00	0.81	0.00	0.00	0.00	0.27	0.19	0.31	0.24
	V	Retinoblastoma	6	1	0	0	1.24	0.00	2.42	0.40	0.00	0.00	0.93	0.68	1.07	0.83
	VI	Renal tumors	9	4	0	2	2.30	0.79	3.63	1.61	0.00	0.72	1.73	1.46	1.93	1.66
	VII	Hepatic tumors	3	0	2	7	0.88	2.78	1.21	0.00	0.79	2.53	0.67	1.17	0.70	1.11
	VIII	Bone tumors	5	7	12	19	4.25	7.54	2.02	2.82	4.73	6.87	3.20	4.19	3.06	3.92
	IX	Soft tissue sarcoma	3	9	2	4	2.48	1.59	1.21	3.62	0.79	1.45	1.87	1.75	1.87	1.77
	X	Germ cell tumors	6	4	8	26	3.19	10.32	2.42	1.61	3.15	9.40	2.40	4.29	2.37	3.95
	XI	Carcinoma & melanoma	8	6	11	41	4.42	16.27	3.23	2.41	4.34	14.82	3.33	6.43	3.29	5.88
	XII	Other & unspecified	22	21	12	34	9.73	13.49	8.88	8.45	4.73	12.29	7.33	8.67	7.54	8.61
		Girls total	207	184	174	252	100.00	100.00	83.60	74.05	68.57	91.07	75.35	79.59	76.15	79.51

a Childhood Cancer Categorized by International Classification of Childhood Cancer, Third Edition, ICCC3.

b The age‐specific incidence (ASR) and crude rate were calculated by per million person‐years.

c The age‐standardized rate was adjusted by World Segi's standardized population (WSR).

**Table 4:** Data quality indicies including MV%, DCO%, and M:F^a^, stratified by areas

ICCC3 category^b^		All			City areas			Rural areas		
		MV%	DCO%	M:F	MV%	DCO%	M:F	MV%	DCO%	M:F
I	Leukemia	98.88	0.56	1.17	98.65	0.00	1.16	98.90	0.61	1.17
II	Lymphoma & related	98.84	0.00	1.52	100.00	0.00	1.01	98.59	0.00	1.67
III	CNS neoplasms	38.51	1.55	1.58	44.44	0.00	1.10	38.16	1.64	1.62
IV	Neuroblastoma	100.00	0.00	1.29	—	—	—	100.00	0.00	1.28
V	Retinoblastoma	84.62	0.00	0.73	100.00	0.00	0.00	83.33	0.00	0.85
VI	Renal tumors	58.97	2.56	1.37	62.50	0.00	2.65	58.06	3.23	1.18
VII	Hepatic tumors	46.43	3.57	1.14	0.00	0.00	—	48.15	3.70	1.07
VIII	Bone tumors	51.22	2.44	1.59	50.00	0.00	0.53	51.30	2.61	1.73
IX	Soft tissue sarcoma	100.00	0.00	1.29	100.00	0.00	0.22	100.00	0.00	1.58
X	Germ cell tumors	79.63	0.00	0.19	40.00	0.00	0.22	83.67	0.00	0.19
XI	Carcinoma & melanoma	100.00	0.00	1.04	100.00	0.00	0.78	100.00	0.00	1.08
XII	Other & unspecified	6.12	10.20	1.03	15.63	0.00	0.78	4.27	12.20	1.09
	Total	74.49	1.80	1.19	73.37	0.00	0.94	74.60	1.98	1.21

a The sex ratio of cancer incidence between boys and girls.

b Childhood Cancer Categorized by International Classification of Childhood Cancer, Third Edition, ICCC3.

### 3.5 Childhood and adolescent cancer incidence stratified by areas

As shown in Table 5, the cancer incidence in rural areas was higher than that in city areas among cases aged 0‐19 years, which were 91.46 and 62.12 per million person‐years, respectively. Among children, cancer incidence in rural and city areas was 88.51 and 62.04 per million person‐years, and the figures were 99.86 and 62.34 per million person‐years among adolescents. Leukemia and CNS neoplasms were the predominant cancer categories in rural area across all age groups. In city areas, leukemia and CNS neoplasms were also the main cancer categories among children; however, leukemia and carcinoma and melanoma were the top two cancer categories among adolescents.

**Table 5:** The incident cases number and incidence rate of childhood cancer during 2010‐2014 in Henan Province, China, stratified by areas

Gender	ICCC3 category^a^		Incident cases number				Proportion (%)		Age‐specific incidence^b^				Crude rate		WSR^c^	
			0‐4	5‐9	10‐14	15‐19	0‐14	15‐19	0‐4	5‐9	10‐14	15‐19	0‐14	0‐19	0‐14	0‐19
City areas	I	Leukemia	19	20	19	16	43.60	31.37	26.27	27.46	27.44	19.56	27.05	24.98	25.22	24.98
	II	Lymphoma & related	1	3	4	7	6.02	13.73	1.38	4.12	5.78	8.56	3.73	5.06	4.92	5.06
	III	CNS neoplasms	5	7	5	1	12.80	1.96	6.91	9.61	7.22	1.22	7.93	6.08	6.27	6.08
	IV	Neuroblastoma	0	0	0	0	0.00	0.00	0.00	0.00	0.00	0.00	0.00	0.00	0.00	0.00
	V	Retino blastoma	1	0	0	0	0.75	0.00	1.38	0.00	0.00	0.00	0.47	0.34	0.35	0.34
	VI	Renal tumors	5	1	2	0	6.02	0.00	6.91	1.37	2.89	0.00	3.73	2.70	2.83	2.70
	VII	Hepatic tumors	1	0	0	0	0.75	0.00	1.38	0.00	0.00	0.00	0.47	0.34	0.35	0.34
	VIII	Bone tumors	0	1	3	4	3.01	7.84	0.00	1.37	4.33	4.89	1.87	2.70	2.62	2.70
	IX	Soft tissue sarcoma	0	4	1	0	3.76	0.00	0.00	5.49	1.44	0.00	2.33	1.69	1.74	1.69
	X	Germ cell tumors	1	1	1	2	2.26	3.92	1.38	1.37	1.44	2.44	1.40	1.69	1.66	1.69
	XI	Carcinoma & melanoma	0	3	5	9	6.02	17.65	0.00	4.12	7.22	11.00	3.73	5.74	5.52	5.74
	XII	Other & unspecified	5	9	6	12	15.00	23.53	6.91	12.36	8.67	14.67	9.33	10.80	10.61	10.80
		City total	38	49	46	51	100.00	100.00	52.55	67.27	66.44	62.34	62.04	62.12	62.09	62.12
Rural areas	I	Leukemia	229	213	191	184	50.10	36.73	48.39	45.33	39.49	36.67	44.36	42.36	42.54	42.36
	II	Lymphoma & related	14	19	19	19	4.12	3.79	2.96	4.04	3.93	3.79	3.64	3.68	3.67	3.68
	III	CNS neoplasms	64	82	84	74	18.20	14.77	13.52	17.45	17.37	14.75	16.12	15.76	15.76	15.76
	IV	Neuroblastoma	3	0	2	0	0.40	0.00	0.63	0.00	0.41	0.00	0.35	0.26	0.26	0.26
	V	Retinoblastoma	11	1	0	0	0.95	0.00	2.32	0.21	0.00	0.00	0.84	0.62	0.65	0.62
	VI	Renal tumors	19	6	4	2	2.30	0.40	4.01	1.28	0.83	0.40	2.03	1.61	1.65	1.61
	VII	Hepatic tumors	5	1	5	16	0.87	3.19	1.06	0.21	1.03	3.19	0.77	1.40	1.36	1.40
	VIII	Bone tumors	7	18	40	50	5.15	9.98	1.48	3.83	8.27	9.97	4.56	5.96	5.83	5.96
	IX	Soft tissue sarcoma	9	12	7	12	2.22	2.40	1.90	2.55	1.45	2.39	1.96	2.07	2.07	2.07
	X	Germ cell tumors	9	5	8	27	1.74	5.39	1.90	1.06	1.65	5.38	1.54	2.54	2.48	2.54
	XI	Carcinoma & melanoma	18	23	25	63	5.23	12.57	3.80	4.89	5.17	12.56	4.63	6.69	6.56	6.69
	XII	Other & unspecified	49	32	29	54	8.71	10.78	10.35	6.81	6.00	10.76	7.71	8.50	8.48	8.50
		Rural total	437	412	414	501	100.00	100.00	92.34	87.68	85.59	99.86	88.51	91.46	91.33	91.46

a Childhood Cancer Categorized by International Classification of Childhood Cancer, Third Edition, ICCC3

b The age‐specific incidence (ASR) and crude rate were calculated by per million person‐years.

c The age‐standardized rate was adjusted by World Segi's standardized population (WSR).

### 3.6 Cancer mortality

A total of 808 cases aged 0‐19 years (582 children, 226 adolescents) died of cancer during 2010‐2014, and the mortality was 36.32 per million person‐years with value in children and adolescents 35.46 and 38.73 per million person‐years, respectively. Among children, it was higher in boys than that in girls with the mortality of 38.48 and 31.87 per million person‐years, and the sex ratio was 1.21. It was also higher in boys among adolescents with the mortality of 41.07 and 36.14 in boys and girls, respectively. Cancer mortality among children and adolescents was all higher in rural areas than that in city areas, with value of 36.09 and 31.25 per million person‐years among children in rural and city areas, respectively, and with the value of 39.66 and 33.00 per million person‐years among adolescents in these two types of areas. The age‐specific mortality was peaked at 15‐19 years group, followed by 5‐9 years, 10‐14 years, and 0‐4 years age groups with the mortality of 38.73, 35.56, 35.45, and 35.38 per million person‐years, respectively.

## 4 DISCUSSION

Our study provided the cancer incidence in children and adolescents stratified by gender, age groups, and geographic areas, and it would help to learn about the burden of cancer in this age group and provide clues for the etiologic studies. This study observed that the overall WSR of cancer incidence among children aged 0‐19 in Henan Province was 87.56 per million person‐years in 2010‐2014, which was among the low incidence areas around the world. A recent study reported the worldwide incidence of childhood cancer during 2001‐2010 using 153 population‐based registries data in 62 countries which constitutes a solid baseline to assess the needs and priorities in the prevention of childhood cancer.^1^ The overall average childhood cancer WSR among the world was 140.6 per million population; however, the cancer incidence was varied by geographic areas, and it was higher in developed countries than in developing countries.^1^ WSRs were more than 150 per million person‐years in areas including some subpopulation in North America and Europe, and Oceania; however, areas including sub‐Saharan Africa, Native American in USA, and in South Asia had WSRs less than 100 per million person‐years.^1^ The incidence reported in our study was in line with that in a previous national study which reported the incidence with 87.1 per million person‐years.^9^ The IARC publication also reported the incidence in China with 131.9 per million person‐years using data from six registries which were all located in big cities in China including Beijing, Dalian, Guangzhou, Hong Kong, Shanghai, and Zhongshan.^10^ However, the registries in Henan Province mostly located in rural areas; hence, the data were suitable for comparable with data in IARC publication.

The worldwide childhood cancer incidence is slightly higher in boys than in girls, and the sex ratio between boys and girls varied from 1.1 to 1.4.^1^ Our data showed that the sex ratio was 1.19, and it was in accordance with previous evidence.^7^, ^16^ It was also varied by age and diagnostic categories. The CNS had the highest sex ratio, followed by carcinoma and melanoma and bone tumors; however, germ cell tumors and retinoblastoma were more common in girls than in boys. This may suggest that the difference exists in the susceptibility between boys and girls, and it also to a large extent reflected true differences in disease occurrence.^17^

The mortality data were fewer reported worldwide or regionally, and the overall mortality in Henan Province was also in line with the national data^9^. In China, mortality had not declined over the past decades, and the 5‐year survival rate was 71.9% which was lower compared to developed countries where 5‐year relative survival rate was commonly more than 80%.^5^, ^16^, ^18^ However, in Henan Province, the survival rate was still not available to estimate, and future work still needs to enrich the epidemiological description.

Leukemia is the most common childhood cancer in children and adolescents in Henan Province regardless of gender and areas, which was in line with previous reports in China^19^ and other areas of the World.^1^ Leukemia accounted for 49.5% of the total cancer among children and 45.74% among adolescents, it was higher than that in Beijing,^19^ and other cities in China where data available including Dalian, Guangzhou, Shanghai, Zhongshan, and Hong Kong.^10^ It was also higher than that in developed countries including America^16^ and Europe.^5^ CNS neoplasms were also the main category in total childhood cancer and stratified by gender and areas, and it accounted for 17.69% of the total childhood cancer. It was higher than that in Beijing, Dalian, Guangzhou, and Hong Kong, however, lower than that in Shanghai and Zhongshan.^10^ It was also lower than that in developed countries including America^16^ and Europe. CNS neoplasms were known to be more common in high‐income areas or countries, which is related to the wide availability of diagnostic facilities.

The etiology of all childhood cancer had been studied for several decades, and it still largely remains unknown, and only a relatively small percentage of them had preventive measures.^20^ Although achievement in environment risk factors was obtained, the progress of the causal association with cancer risk determining remains slow due to several reasons including the rarity of childhood cancer, and difficulty in accurate exposure assessment.^8^, ^20^ According to the current evidence, high dose of ionizing radiation is the documented risk factor for cancer in children and adolescents, which increasing risk several fold.^20^, ^21^ Exposures to pesticides and extremely low‐frequency magnetic field were showed to increase the risk of leukemia which is the most common cancer diagnosed in children.^22^, ^23^ Genetic predisposition played important role in the cause of childhood cancer; however, it only accounted for 5% in the development of CNS tumors mainly associated with familial cancer syndromes.^24^ Although efforts had been undertaken to investigate the association between genetic and environmental factors and childhood cancer risk of decades, identification of modifiable risk factors for implementing primary prevention remains the ultimate goal, and further epidemiological studies are still needed to be facilitated to understand the risk factors on childhood cancer in future.

Limitations should be considered in interpreting the results in our study. Firstly, childhood and adolescent cancer mortality was estimated from the vital statistic data, in which cancer cannot be reclassified according to the criteria of ICCC3 due to the information on morphology and behavior were not included. Secondly, the proportion of unspecified subcategory in each main ICCC3 cancer category was relatively high, and the reasons may be including the limitation in diagnostic ability and quality in data collecting and coding in each cancer registry.

In conclusion, this is the first study that described the cancer incidence among children and adolescents in Henan Province, and it would help researchers to understand the burden and epidemiological characteristics of childhood cancer and hence suggested clues for the etiological studies.

## CONFLICTS OF INTEREST

The authors declared no potential conflicts of interest with respect to the research, authorship, and/or publication of this article.
